# Lidar observations of large-amplitude mountain waves in the stratosphere above Tierra del Fuego, Argentina

**DOI:** 10.1038/s41598-020-71443-7

**Published:** 2020-09-03

**Authors:** N. Kaifler, B. Kaifler, A. Dörnbrack, M. Rapp, J. L. Hormaechea, A. de la Torre

**Affiliations:** 1 Institute of Atmospheric Physics, German Aerospace Center, Oberpfaffenhofen, Germany; 2 Estación Astronomica Rio Grande, Facultad de Ciencias Astronomicas y Geofisicas, Universidad Nacional de La Plata & CONICET, La Plata, Argentina; 3 CONICET/Facultad de Ingeniería, Universidad Austral, LIDTUA (CIC), Buenos Aires, Argentina

**Keywords:** Atmospheric science, Atmospheric dynamics

## Abstract

Large-amplitude internal gravity waves were observed using Rayleigh lidar temperature soundings above Rio Grande, Argentina ($54^\circ \; \hbox {S}$, $68^\circ \; \hbox {W}$), in the period 16–23 June 2018. Temperature perturbations in the upper stratosphere amounted to 80 K peak-to-peak and potential energy densities exceeded 400 J/kg. The measured amplitudes and phase alignments agree well with operational analyses and short-term forecasts of the Integrated Forecasting System (IFS) of the European Centre for Medium-Range Weather Forecasts (ECMWF), implying that these quasi-steady gravity waves resulted from the airflow across the Andes. We estimate gravity wave momentum fluxes larger than 100 mPa applying independent methods to both lidar data and IFS model data. These mountain waves deposited momentum at the inner edge of the polar night jet and led to a long-lasting deceleration of the stratospheric flow. The accumulated mountain wave drag affected the stratospheric circulation several thousand kilometers downstream. In the 2018 austral winter, mountain wave events of this magnitude contributed more than 30% of the total potential energy density, signifying their importance by perturbing the stratospheric polar vortex.

## Introduction

The Tierra del Fuego archipelago at the southern tip of South America is known as the world’s gravity wave hot spot^1^. Here, strong tropospheric winds excite mountain waves year-long. In austral winter, the westerlies of the polar night jet (PNJ) allow these mountain waves to propagate deeply into the middle atmosphere where they deposit their momentum and decelerate the mean stratospheric flow^2–4^. So far, mainly satellite-based instruments are utilized for estimations of the stratospheric gravity wave momentum that is transported vertically^5–16^. Due to the observational filter of these instruments, only a part of the full wave spectrum is included. This leads to an underestimation of the fluxes that are significant input parameters for global circulation models^17–20^. Therefore, our lead question is: can local, continuous and high-temporal- and high-vertical-resolution observations of gravity waves in the lee of the Andes enhance our knowledge about the magnitude of the momentum that is deposited in the stratosphere? Such observations can be accomplished using powerful, vertically-pointing Rayleigh lidars as operated at few locations world-wide^21–27^. To specifically target the worlds’s largest gravity waves, we installed the Compact Autonomous Rayleigh lidar (CORAL) at Rio Grande, Tierra del Fuego, Argentina, for night-time measurements of atmospheric temperature. Here, we will present a case study of an exceptionally strong mountain wave event with peak-to-peak temperature amplitudes up to 80 K and a long duration of eight days during austral winter 2018.

Gravity waves are known to be highly intermittent, resulting in occasional but large-amplitude events that have the potential to dominate the total momentum budget^28^. Stratospheric gravity waves with peak-to-peak amplitudes of 10–30 K were observed directly above the Andes mountains, Rothera or South Georgia island^14,15,29–32^. Satellite records revealed a leeward extension of momentum flux to the Atlantic Ocean, in combination with generally increased values at the $60^\circ \;\hbox {S}$ latitude band, whose origin has been subject to debate. Simulations with a gravity wave-resolving model showed that mountain waves from the southern Andes can propagate several thousand kilometers downstream^33^. The lidar measurements we present here will retrace the evolution of the seasons’s strongest event during the course of several days. The observations of temperature perturbations, momentum flux and gravity wave drag will be complemented with and compared to data from an operational numerical weather prediction (NWP) model. We will discuss the excitation and downstream advection of the mountain waves using model data. The seasonal evolution of the stratospheric gravity wave activity above Rio Grande will be documented based on observations and NWP data. The CORAL lidar’s long-term dataset with dense temporal coverage will be exploited to assess the contributions of mountain wave events with similar magnitude to the total gravity wave activity during austral winter 2018.

## Results

### Observations and IFS data of temperature and wind

**Figure 1:**
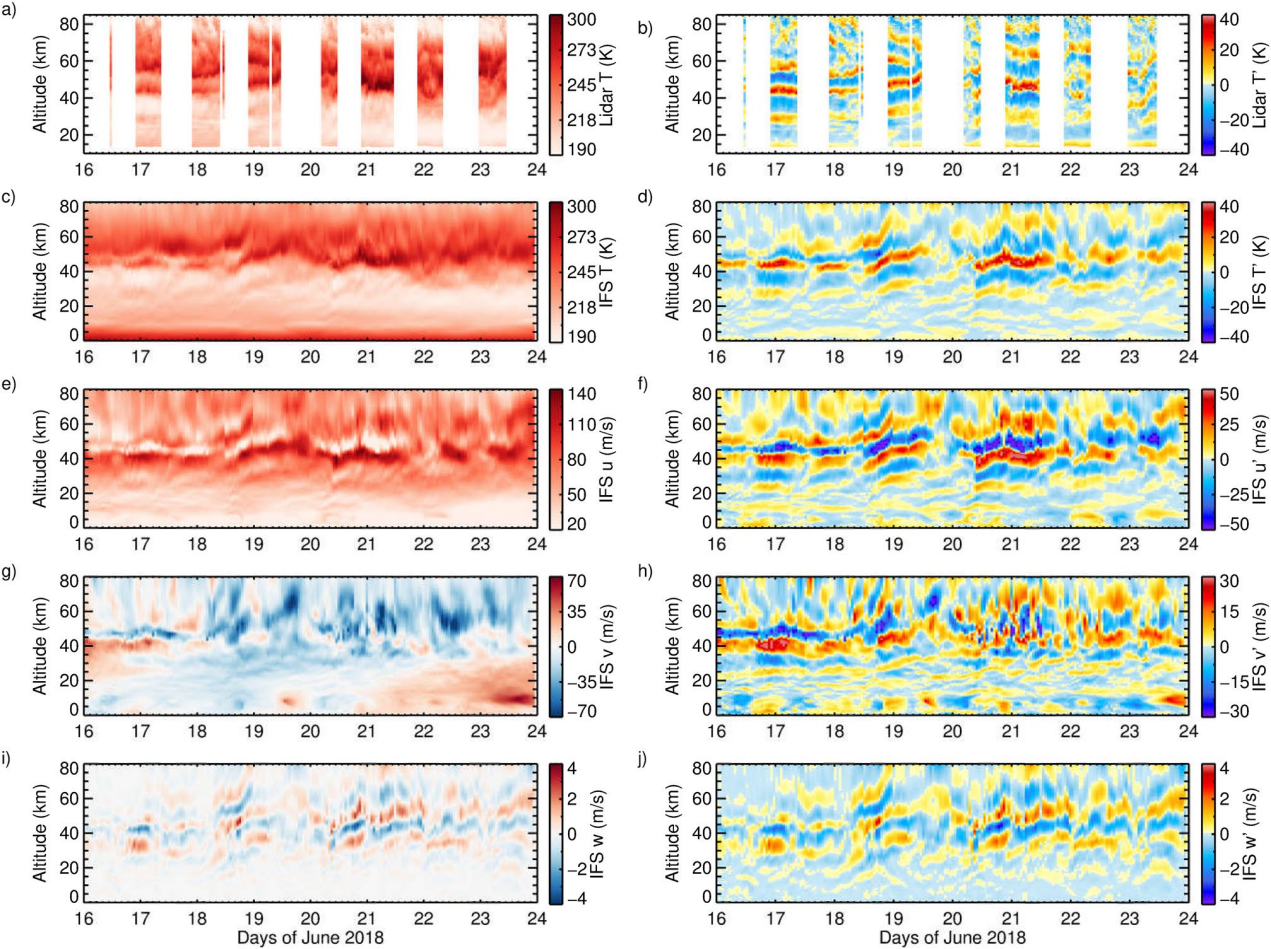
Altitude-time sections of (**a**,**b**) lidar temperature *T* and temperature perturbations $T'$, (**c**,**d**) IFS temperature *T* and temperature perturbations $T'$, (**e**,**f**) IFS zonal wind *u* and perturbations $u'$, (**g**,**h**) IFS meridional wind *v* and perturbations $v'$, and (**i**,**j**) IFS vertical wind *w* and perturbations $w'$ above Rio Grande, Argentina, from 16–23 June 2018 at 1 h resolution between 0 or 15 and 85 km altitude.

The CORAL lidar instrument and data analysis are described in the “Methods” section below. Night-time lidar temperature measurements between 15–85 km altitude above Rio Grande in the period 16–23 June 2018 are presented in Fig. 1a. As expected from the climatological mean, the temperature maximizes between 40–70 km where the dynamically induced winter stratopause is located. The vertical temperature profiles in this stratopause region are, however, dominated by up to three temperature maxima due to the impact of mountain waves. The associated temperature perturbations reveal coherent phase patterns with peak-to-peak amplitudes up to 80 K (Fig. 1b). The wave signatures exist throughout the observed altitude range and their amplitudes are largest between 40–55 km. The altitude of the phases with maximum amplitude is predominantly constant in time; however, there are nights when the phases slowly ascend or descend in time. Overall, we conclude that these are signatures of quasi-stationary gravity waves, i.e. mountain waves. In the eight-day mean (not shown), temperature maxima occur at 29 and 45 km altitude. This implies a mean vertical wavelength of 16 km, that varies between 12 and 20 km during particular nights. Vertical temperature gradients between 38 km and 48 km are very close to the adiabatic lapse rates, indicating possible mountain wave overturning. A plot of potential temperature is shown in Supplementary Fig. S1.

The thermal structure measured by the lidar is very well reproduced by the IFS (Fig. 1c) with only minor differences. Temperature perturbations (Fig. 1d) are strikingly similar to the lidar observations, however, the descending phase lines observed above 60 km altitude on 18 and 19 June are absent in the IFS. This means that only upward propagating waves are simulated by the IFS and their amplitude fades with height due to the numerical damping applied. The wave amplitude in the IFS is strongest between 30 and 60 km. Temperature perturbations up to 75 K are attained and the vertical wavelength matches the lidar observations. During this event, also the wave phases observed by the lidar and predicted by the IFS coincide in altitude and duration. Analyses of other events have not always resulted in such good agreement. It should be noted that the temperature perturbations of the CORAL observations and the IFS are calculated by two different methods for determining the ambient temperature profiles. The astonishing coincidence provides confidence in utilizing the 3D IFS fields for determining horizontal wavelengths of the dominating mountain waves.

Figure 1e–j show the IFS zonal, meridional and vertical wind and the corresponding perturbations above Rio Grande for the same period. The zonal wind associated with the PNJ is large in the lower stratosphere. Yet, the wind field is disturbed by mountain waves in the upper stratosphere leading to an enhanced vertical shear at about 40 km altitude (Fig. 1e). The flow is from the west with a weaker southward component that changes to a northward component around 17 June in the lower stratosphere, likely caused by planetary waves meridionally displacing the polar vortex edge (Fig. 1g). The phases and vertical wavelengths of the zonal wind field match those seen in the thermal structure. The average (maximum) peak-to-peak perturbations are 56 m/s (157 m/s at 4 UT on 21 June). Due to phase shifts in altitude this value is lower for the mean zonal wind profile for 16–24 June, but is still 23 m/s between 41.5 and 47.5 km.

Peak-to-peak perturbations are 19 m/s in the mean and 150 m/s at one hour resolution (Fig. 1f). In the mean, the zonal wind is accelerated at 40 km altitude and decelerated at 51 km altitude, which leads to a vertical gradient of − 7 m/s/km. At 1 h resolution, the absolute vertical gradients are as large as 67 m/s/km. The perturbations in meridional wind show wave-like behaviour similar to the zonal wind perturbations, but exhibit high variability on 20/21 June when the zonal wind is strongly decelerated (Fig. 1h). Maximum perturbation amplitudes of the vertical wind are 3.2 m/s (Fig. 1j).

### Momentum flux and gravity wave drag

The vertical flux of horizontal momentum, abbreviated as gravity wave momentum flux *F*, can be estimated from temperature as^6^

1 $$\begin{aligned} \vec {F_T} = \rho E_p \frac{\vec {k}}{m} \end{aligned}$$

with the potential energy density

2 $$\begin{aligned} E_p = \frac{1}{2}\frac{g^2}{N^2} \overline{\left( \frac{T'}{T_0}\right) ^2}. \end{aligned}$$

The atmospheric density $\rho = \frac{p_0}{R T_0}$ is calculated from synoptic IFS pressure $p_0$ and temperature $T_0$. *R* denotes the gas constant and *g* the acceleration due to gravity. $T, T', T_0$ and the squared Brunt-Vaisälä frequency $N^2 = \frac{g}{T} (\frac{dT}{dz} + \frac{g}{c_p})$ are taken from night-time lidar measurements or IFS, respectively, as described in the “Methods” section below. The overline indicates a root mean square average over a vertical range of 16 km, corresponding to the mean vertical wavelength. The horizontal and vertical wavenumbers $k = \frac{2 \pi }{\lambda _h}$ and $m = \frac{2 \pi }{\lambda _z}$ are calculated from the respective horizontal or vertical wavelengths. We estimated $\lambda _h$ from latitude–longitude IFS maps of $T'$ between 40 and 55 km altitude, finding $\lambda _h = 260\,\pm \,100~\hbox {km}$ in predominantly zonal direction and, for simplicity and comparability, use this mean value for all calculations. For $\lambda _z$ we use the lidar mean value of $16 \pm 3~\hbox {km}$.

In order to validate experimentally the assumptions used by deriving Eq. (2), we also computed the total momentum flux from IFS wind perturbations using

3 $$\begin{aligned} |{F}_{u,v}| = \sqrt{F_x^2 + F_y^2} = \sqrt{(\rho \overline{u' w'})^2 + (\rho \overline{v' w'})^2}. \end{aligned}$$

**Figure 2:**
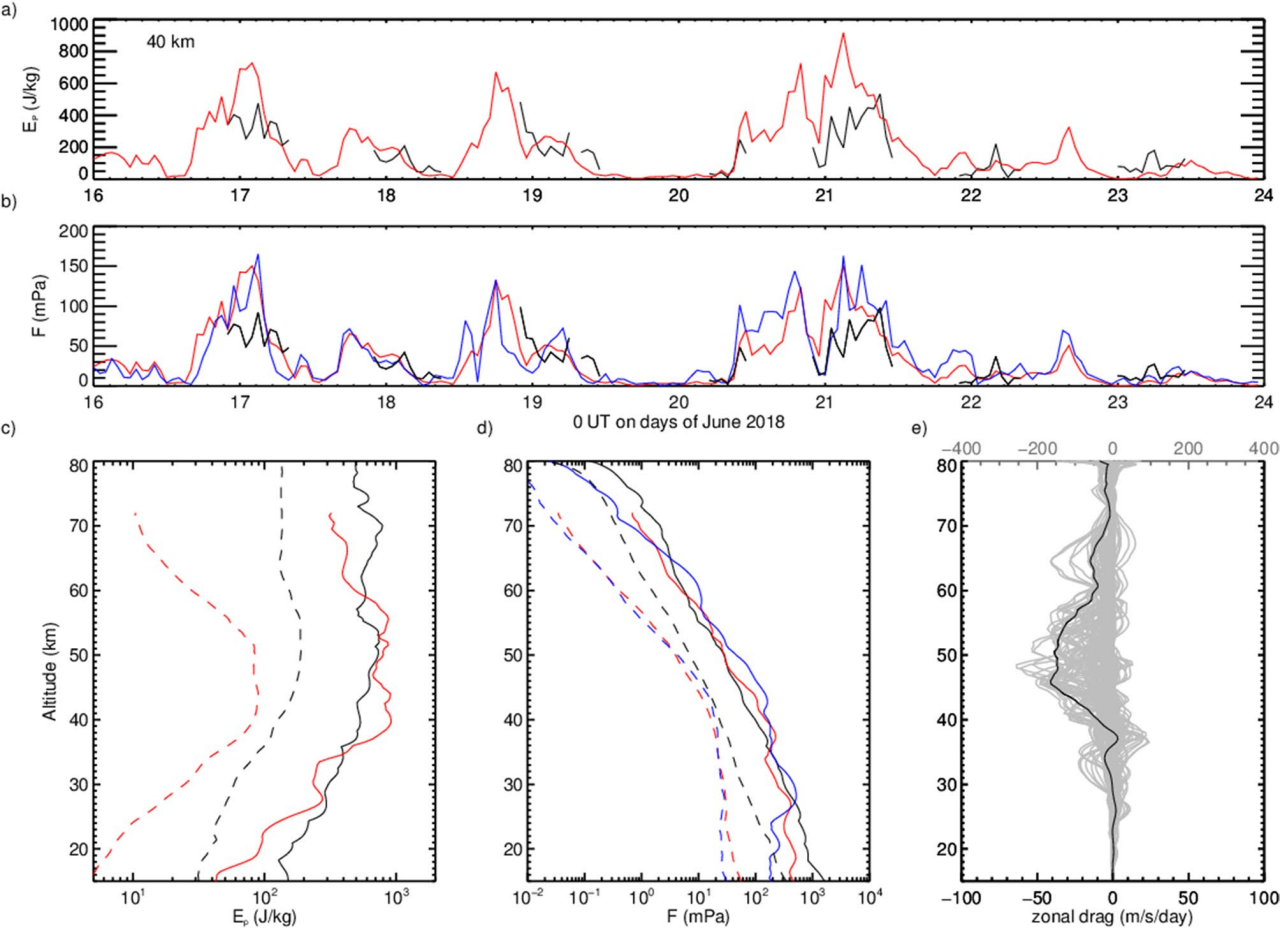
Time series of (**a**) potential energy density $E_p$ derived from lidar $T'$ (black) and IFS $T'$ (red), and (**b**) derived absolute momentum fluxes *F* including $F_{\mathrm {u,v}}$ (blue) at 40 km altitude. Vertical profiles of mean (dashed) and peak (solid) (**c**) $E_p$ and (**d**) *F* in the same colors. (**e**) Mean zonal drag (black, bottom axis) and a set of 12-h averages obtained by shifting the interval middle by two hours (grey, top axis with larger scale). The drag was calculated from $F_x = \rho \overline{u' w'}$.

The temporal evolution of $E_p$ and *F* at 40 km altitude are displayed in Fig. 2a,b. The $E_P$-values at 40 km altitude reveal an oscillatory behaviour during the considered period. This indicates that pulses of individual mountain waves reach the stratosphere and the entire large-amplitude mountain wave event consisted of a succession of upward propagating gravity waves in accordance with the temporal evolution of the tropospheric winds (see Supplementary Fig. S1). The same temporal enhancements and decays are also visible in the momentum flux curves. All curves show similar absolute values and temporal evolutions across the three methods at most times. Differences might arise due to variations of $\lambda _h$ and $\lambda _z$. The logarithmic mean, the arithmetic mean and peak values are 23 mPa, 33 mPa and 100 mPa for $F_{\mathrm {T,lidar}}$, 11 mPa, 27 mPa and 150 mPa for $F_{\mathrm {T,IFS}}$, and 14 mPa, 28 mPa and 165 mPa for $F_{u,v}$.

Figure 2cd show vertical profiles of mean and peak $E_p$ and *F*. $E_p$ increases up to 40–55 km, and above, *F* decreases more rapidly, indicating a limitation of the mountain wave’s amplitude. The zonal wave drag is given by the vertical divergence of the zonal momentum flux

4 $$\begin{aligned} \frac{\partial u}{\partial t} = - \frac{1}{\rho } \frac{\partial }{\partial z} F_x. \end{aligned}$$

The mean drag is negative between 38 km and 70 km altitude and maximizes around 48 km with values of $\approx - 40~ \hbox {m/s/day}$ (Fig. 2e). Shown in light grey are a set of 12 h-average profiles of the zonal wave drag that exhibit significantly larger absolute values up to 200 m/s/day between 40–55 km altitude, including a secondary maximum in the mid mesosphere.

### Excitation and downwind propagation

Analyses of IFS tropospheric zonal wind at an upstream location north-west of Rio Grande ($50^\circ \; \hbox {S}$, $285^\circ \; \hbox {E}$) show enhanced wind speeds between 15 and 21 June 2018. Mean (maximum) zonal wind speeds are 18 m/s (28 m/s) at 2 km altitude in this time period, see Supplementary Fig. S2. Weaker southward meridional winds of about 10 m/s until 20 June result in a steady flow orthogonal to the Andes mountain range. Therefore, strong forcing of mountain waves above the southern Andes occurred during a period of several days. Strong zonal jet streams of up to 67 m/s at 10 km altitude and eastward winds in the lower stratosphere, most strongly on 21/22 June, facilitated the propagation of the excited waves into the upper stratosphere.

**Figure 3:**
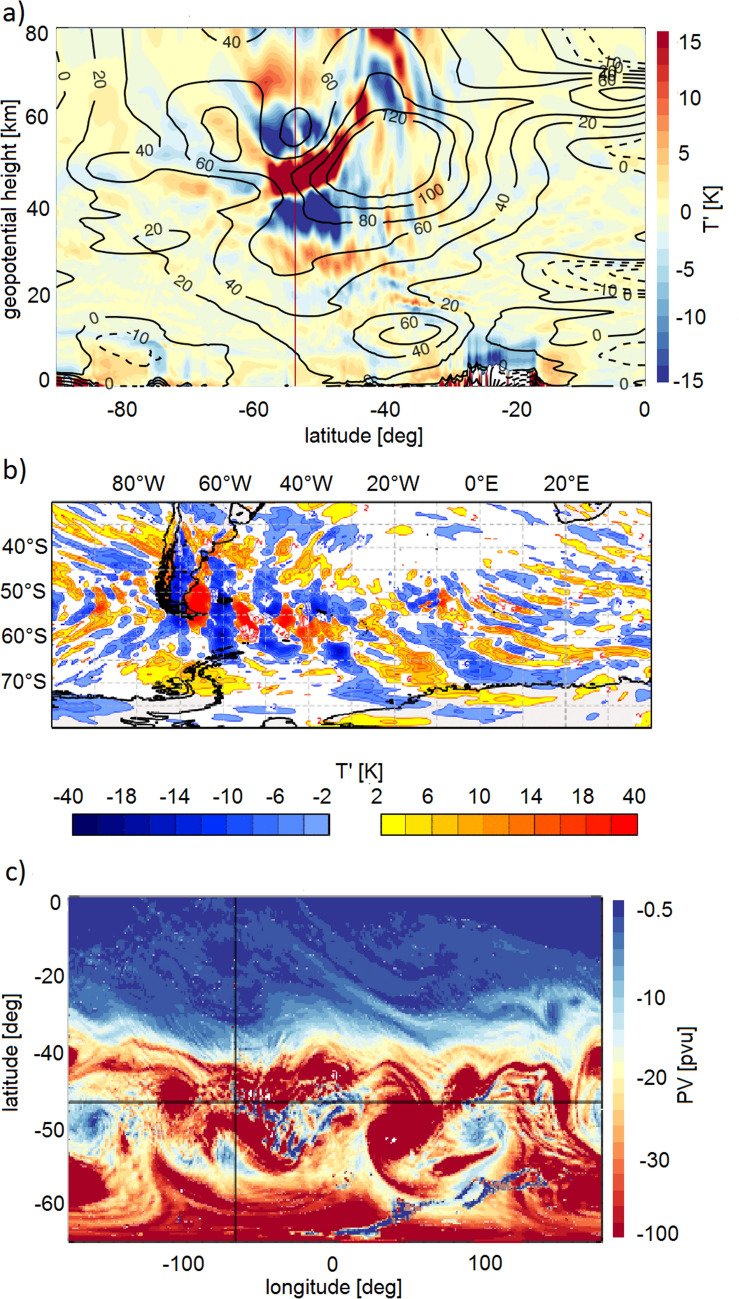
(**a**) Altitude-latitude section of IFS $T'$ (color) and zonal wind (contours) at the longitude of Rio Grande, (**b**) map of IFS $T'$ at 40 km altitude, and (**c**) map of potential vorticity at 2,000 K potential temperature ($47 \pm 7~\hbox {km}$), all for 21 June 2018 at 0 UT. The cross in (**c**) marks the position of Rio Grande.

Further analyses of latitude–altitude sections of zonal wind and temperature perturbations above Rio Grande show the polar vortex to be tilted northwards in the stratosphere and spanning the latitude band from $60^\circ \; \hbox {S}$ to about $30^\circ \; \hbox {S}$ (Fig. 3a and Supplementary Fig. S3). The core of the PNJ with $u > 120~\hbox {m/s}$ was located north of the observation site at about $35{-}40^\circ \; \hbox {S}$ at 50–60 km altitude between 14–23 June. On 14 June, forcing conditions at about 2 km altitude were too weak for the excitation of mountain waves. Additionally, weak stratospheric winds might have attenuated a possible vertical propagation. The situation changed on 16 June when the polar front jet shifted northwards to the latitude of Rio Grande, strengthened, and merged with the subvortex jet as indicated by increased wind speeds in the lower stratosphere^34^. This process allowed for the vertical propagation of mountain waves inside the polar vortex edge to 45 km altitude, where amplitudes maximized at the inner edge of the PNJ core. The momentum deposition by the mountain waves above 50 km altitude accounted for a strong deceleration of the zonal wind, deforming the polar vortex with a tendency to develop a secondary wind maximum south of Rio Grande. The merged subvortex jet slowly moved northward, and the continuous excitation of mountain waves and the nearly stationary location of the PNJ core led to refraction of the mountain waves to the upper stratosphere above Rio Grande for a duration of 8 days.

Thus a large mountain wave drag acted continuously on the stratospheric circulation for a 1-week period and affected the stratospheric flow downstream of Tierra del Fuego. Figure 3b presents an IFS $T'$ map of the larger geographic area for 0 UT on 21 June 2018. Clear signatures of mountain waves originating from the ridge of the southern Andes to the north-west of the lidar station are visible in Fig. 3b. The mountain wave pattern extended more than 1500 km in north-south direction, and several $T'$-minima and maxima were visible leeward to at least $30^\circ \; \hbox {W}$. Figure 3c shows a map of potential vorticity (PV) at the 2,000 K isentrope (about 47 km altitude). Due to diabatic processes (frictional drag of the dissipating mountain waves), negative PV was produced and transferred to the background flow, visible by smaller-scale structures of blue color emanating eastwards from the observation site within the polar vortex edge. The disturbance reached from Tierra del Fuego to about $20^\circ \; \hbox {E}$, which corresponds to a horizontal distance of 6,000 km. Daily snapshots of temperature, wind and PV are shown in the Supplementary Figs. S3 and S4.

## Discussion and summary

Increased momentum fluxes leeward of the Andes have been correlated with rare, large-amplitude orographic waves based on satellite data as well as model simulations^13,33^. Our measurements of mountain wave-induced temperature perturbations of 80 K are a factor of two or more larger than the strongest events observed by satellite instruments with amplitudes in the range 10–30 K^1,14,15,29,30,32^. However, a large-amplitude mesospheric mountain wave with 67 K peak-to-peak amplitude was observed by ground-based lidar for a short duration of 2 h above Lauder, New Zealand, which is also a gravity wave hot spot^35,36^. At southern high latitudes, intra-annual and seasonal gravity wave statistics are available from a number of lidar stations in Antarctica^22–25^. They all indicate increased gravity wave activity during winter, but likely do not capture the strongest events due to their location within the polar vortex. The observed long vertical wavelength of 16 km is in accordance with estimates from linear theory and the large zonal winds during the events as computed by the IFS. Furthermore, the value is in general agreement with climatologies yielding mean wavelengths of 12–14 km and single events of e.g. 20 km^1,32^. Observed momentum fluxes above 100 mPa are much larger than monthly mean fluxes of 1–3 mPa at 40 km derived from satellite data above Tierra del Fuego during July and August^1,10,13,16,19^. High-resolution analyses of Atmospheric Infrared Sounder (AIRS) data however yielded comparable or larger values^14,15,30^. The mean gravity wave drag in the upper stratosphere derived from satellite data at $50^\circ \; \hbox {S}$ in July amounts to 5–10 m/s/day^10^. In the zonal mean, the gravity wave drag increases with altitude. As shown in this case study, the drag excerted in the upper stratosphere during such extreme events can be a factor of ten larger for several days, and even larger for shorter periods of 12 h. The duration of the mountain wave event described in the current study was significantly longer than previously described episodes (24 h or 4 days^30,32^). This prolonged duration in combination with high momentum fluxes and large gravity wave drag in the upper stratosphere has profound influence on the stratospheric circulation as documented by the IFS PV field in Fig. 3c.

**Figure 4:**
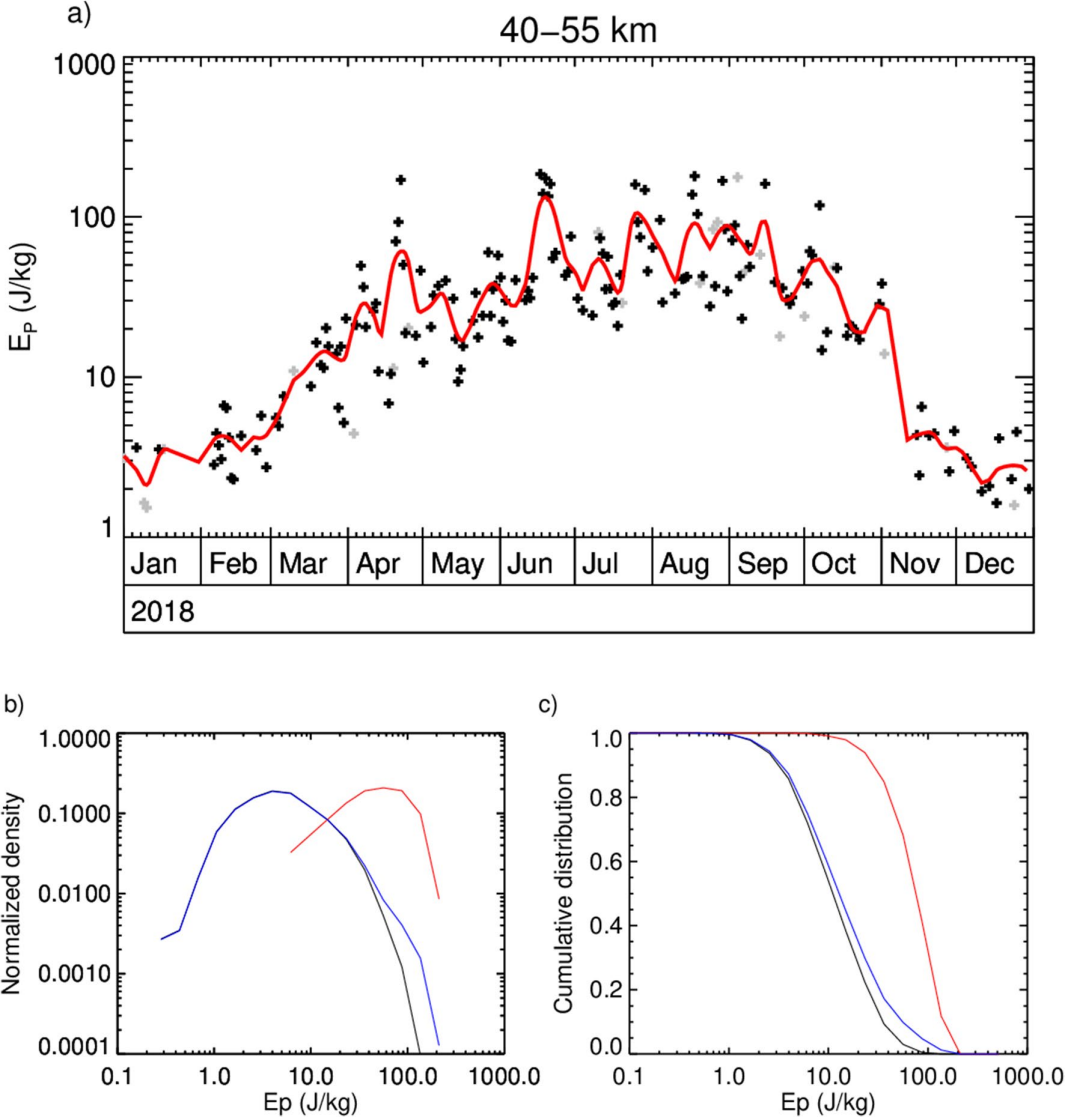
(**a**) Seasonal evolution of nightly $E_p$ averaged between 40 and 55 km altitude as measured by CORAL above Rio Grande in 2018. Grey dots mark datasets shorter than 3 h. The red line represents the data filtered by a 15 day Hann filter. (**b**) Normalized distribution of the 15 nights with highest $E_p$ (red), all winter values (April–September, blue), and the ones excluding the nights of the strongest 15 events (black). (**c**) Corresponding cumulative distributions.

The relative importance of an event of this magnitude not only depends on its amplitude and duration, but also on its occurrence rate or intermittency. In Fig. 4a we show the seasonal evolution of nightly mean $E_p$-values averaged between 40 and 55 km altitude based on CORAL lidar soundings with 20 min temporal and 900 m vertical resolution and a vertical filter with a 15 km cut-off wavelength. Increased $E_p$ during winter conditions is observed from April to October 2018 with a seasonal average of 52 J/kg. Although absolute values delicately depend on the altitude range, resolution and spectral filtering of measured temperature profiles, this value is larger than corresponding values obtained from satellite soundings for the same geographical area and other lidar measurements from stations in Antarctica^1,15,22–25^. Latter measurements were conducted most likely well within the polar vortex and thus do not detect the maximum $E_p$-values that are specific to the polar vortex edge^7,21,37,38^. From the station in Rio Gallegos, Argentina, only gravity wave amplitudes for the lower stratosphere are available^27^. The high intermittency of gravity waves in winter appears in the high variability of $E_p$ as shown in Fig. 4a. In this regard, the multi-day mountain wave event of June 2018 clearly stands out among other events of shorter duration but similar magnitude. This shows that although the duration of the presented event may be exceptional and nightly mean $E_p$ values are more than three times the seasonal average, it is not the only strong event of the season. $E_p$ is above twice the winter mean for 15 nights which account for 8% of the winter dataset: 22 April, 17–21 June, 25 and 29 July, 17–19 and 29 August, 4 and 15 September, and 7 October. The dates refer to the morning, i.e. 7 October is to mean the night from 6 to 7 October. In order to assess the contribution of these strongest events, in Fig. 4b,c we juxtapose the normalized and cumulative distributions of all winter $E_p$ values (blue), of the subset of the named 15 nights with the strongest events (red), and of the subset excluding these events (black) between 40 and 55 km. The distribution of the selected nights with the strong events peaks at a value about 15 times higher than the distribution of all winter observations. That is, 8% of the dataset exhibit about 15 times higher $E_p$ than the seasonal mean. Accumulating all winter $E_p$ values, those selected strong events contribute more than 30% to the total $E_p$.

In summary, we have performed local, high-resolution night-time lidar soundings at the location of the world’s strongest gravity wave hotspot above the southern tip of South America. Large-amplitude mountain waves were observed in the upper stratosphere during the period 16–23 June 2018. The gravity wave-induced temperature perturbations of 80 K exceed the largest so far reported values derived from satellite observations and are associated with high potential energy densities, high momentum fluxes and large gravity wave drag. The event is very well reproduced by IFS data in terms of amplitude and phase of the waves. IFS analyses show that the mountain waves were continuously excited by a latitudinally shifting polar front jet associated with strong tropospheric zonal winds orthogonal to the Andes mountains. Presumably, such conditions are similar to the sustained mountain waves in the lee of the Andes that are known to glider pilots and allow for record-long meridional flights in that region. The mountain waves propagated upwards supported by the increased zonal winds in the polar vortex and were refracted towards the inner edge of the PNJ. There, they induced strong wind shears and, due to the prolonged duration of the event, constituted a profound disturbance of the stratospheric circulation extending several thousand kilometers leewards towards the Atlantic Ocean.

Analysis of this event and similar shorter-lived large-amplitude mountain wave events during winter 2018 has shown that quasi-stationary mountain waves account for a substantial fraction of the accumulated potential energy densities in the stratosphere and lower mesosphere. They thus have the potential to contribute significantly to the increased momentum flux leeward of the Andes that is observed in satellite data and model simulations. Moreover, this case study demonstrates the benefit of autonomous, high-resolution, ground-based Rayleigh lidar observations. The dense data coverage not only facilitates the detailed understanding of single high-impact events, but also helps with putting global observations at coarser resolution into a context.

## Methods

### Lidar

The CORAL lidar has been developed and built at DLR. It is the first of a new class of high-power, autonomously operating lidars allowing for high vertical and high temporal resolution measurements of atmospheric density during night-time. Data from CORAL and its twin TELMA obtained during several multi-month campaigns in both hemispheres have been used for studies of gravity waves and other phenomena of the mesosphere like tides and noctilucent clouds^39–43^. The Rayleigh lidar emits 12 W power at 532 nm wavelength and receives backscattered photons with a 63 cm diameter telescope using three height-cascaded elastic detector channels and one Raman channel. CORAL is a portable lidar and commenced operation at Estación Astronómica Río Grande ($53.78^\circ \; \hbox {S}$, $67.75^\circ \; \hbox {W}$) at the east coast of Tierra del Fuego in November 2017, where it was a part of the international network of complementary instruments dedicated to the study of middle atmosphere dynamics within the ARISE (Atmospheric Dynamics Research InfraStructure in Europe) project^44^. CORAL operates autonomously during clear sky conditions in darkness. Weather conditions are continuously and automatically assessed based on local observations and short-term IFS forecasts of clouds and precipitation. During two years of operation, 2,450 h of high-quality data were collected.

Nightly mean temperatures are derived by hydrostatic integration of the measured density profiles^45^, using a seed temperature at 100–110 km altitude obtained from SABER. The procedure is repeated for higher-resolution profiles of 2 h and then 1 h integration time using the previously obtained coarser profiles for seeding. For this case study, we use temperature profiles with 1 h temporal and 500 m vertical resolution between 15 and 80 km altitude. Errors in retrieved absolute temperatures strongly decrease with progress of the downward integration and amount to $\sim$4 K at 80 km altitude and $\sim$0.5 K at 55 km altitude. Vertical temperature profiles *T*(*z*) are decomposed into the background temperature $T_0(z)$ and the temperature perturbation $T'(z)$ attributed to gravity waves using a 5th order Butterworth spectral filter^46^. A vertical cut-off wavelength of 20 km was used in order to include mountain waves with long vertical wavelengths. During the mountain wave event on 16–23 June 2018, for the most part excellent weather conditions allowed for operation of the lidar for a total of 80 h.

### IFS

Lidar data were complemented with operational analyses and short-term deterministic forecasts of the Integrated Forecasting System (IFS) of the European Centre for Medium-Range Weather Forecasts (ECMWF)^47^. The IFS is a global, hydrostatic, semi-implicit, semi-Lagrangian model for numerical weather prediction. One-hour resolution IFS temperatures *T*, zonal winds *u*, meridional winds *v* and vertical winds *w* were interpolated to the location of Rio Grande and to 500 m vertical intervals from the surface to 80 km altitude. Perturbations of temperature and wind, denoted as $T', u', v'$ and $w'$, were inferred by subtraction of *T*, *u*, *v*, *w* retrieved at reduced spectral resolution of wavenumber 21 as $f' = f_{\mathrm {CO1279}} - f_{\mathrm {CO21}}$, where $f={T,u,v,w}$.

$T_{\mathrm {CO1279}}$ denotes fields with ${\widehat{=}}$ 9 km horizontal resolution and $T_{\mathrm {CO21}}$ fields with ${\widehat{=}} 1000~\hbox {km}$ horizontal resolution. The resulting perturbations show gravity waves and exclude synoptic-scale features like planetary waves and inertial instabilities^48, 49^. Gravity waves are well represented in IFS analyses up to an altitude of 55 km^50^. At higher altitudes, simulated wave activity is damped within the model’s sponge layer for numerical reasons.

## Supplementary information

Supplementary Information.
