# Supplementary material for: Lidar observations of large-amplitude mountain waves in the stratosphere above Tierra del Fuego, Argentina
Source: Sci Rep. 2020 Sep 3;10:14529. doi: 10.1038/s41598-020-71443-7 (PMC7471968; doi:10.1038/s41598-020-71443-7)
Supplement: Supplementary file 1 — Supplementary Information. [file 41598_2020_71443_MOESM1_ESM.pdf]

Supporting information for „Lidar observations of large-amplitude mountain waves in the stratosphere above Tierra del Fuego, Argentina"

N. Kaifler[1,*], B. Kaifler[1], A. Dörnbrack[1], M. Rapp[1], J. L. Hormaechea[2], and A. de la Torre[3]

[1]Institute of Atmospheric Physics, German Aerospace Center, Oberpfaffenhofen, Germany
[2]Estación Astronomica Rio Grande, Facultad de Ciencias Astronomicas y Geofisicas, Universidad Nacional de La Plata & CONICET, Argentina
[3]CONICET / Facultad de Ingeniería, Universidad Austral, LIDTUA (CIC), Argentina
[*]natalie.kaifler@dlr.de

ABSTRACT

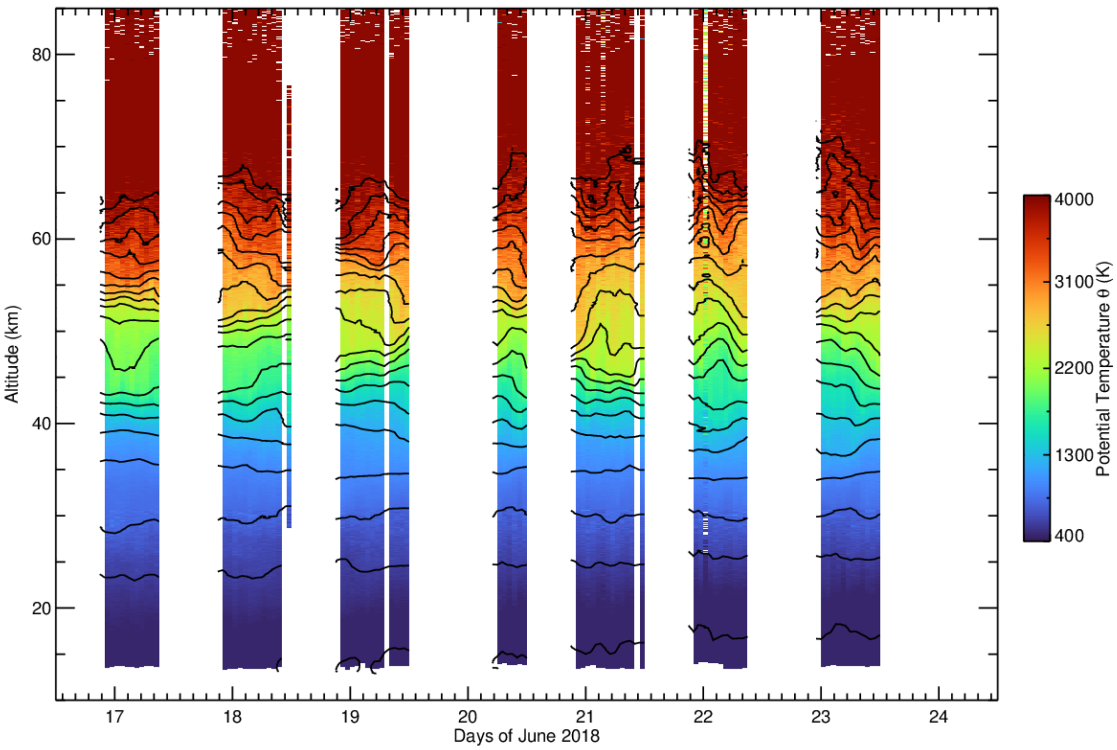

Figure S1. Potential temperature calculated from measured lidar temperature and density. The contour lines are plotted every 200 K.

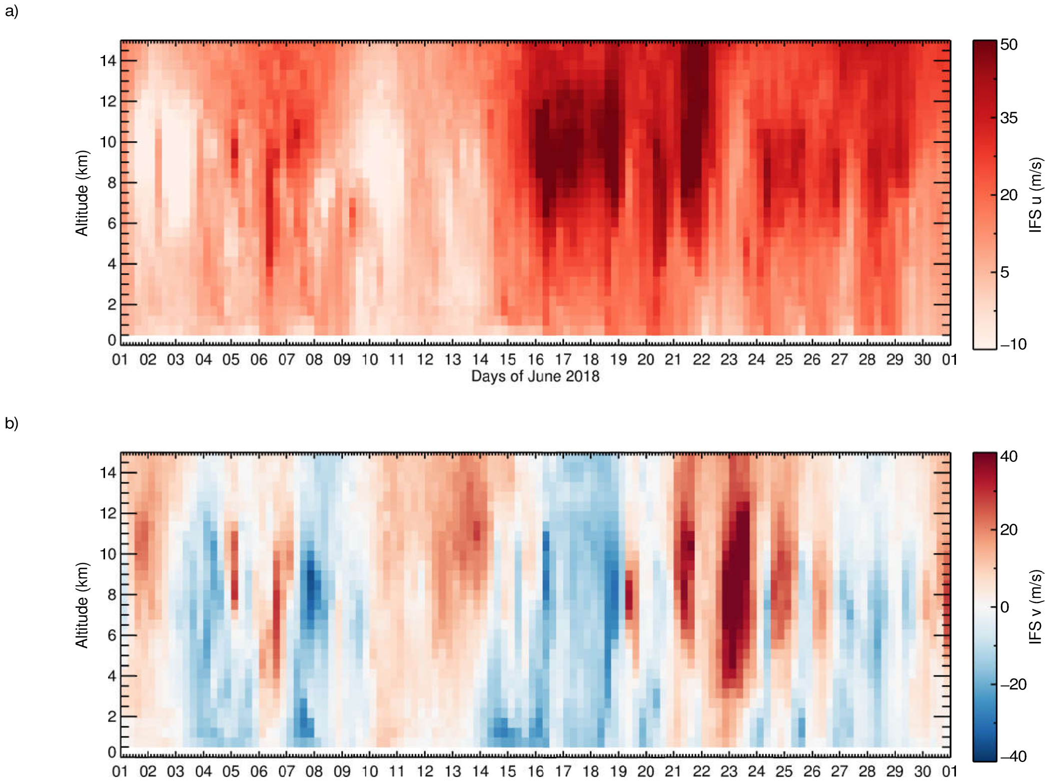

Figure S2. Altitude-time sections of tropospheric (a) zonal and (b) meridional wind from the IFS in June 2018 at 6 h resolution for an upstream position north-west of Rio Grande (50°S, 285°E).

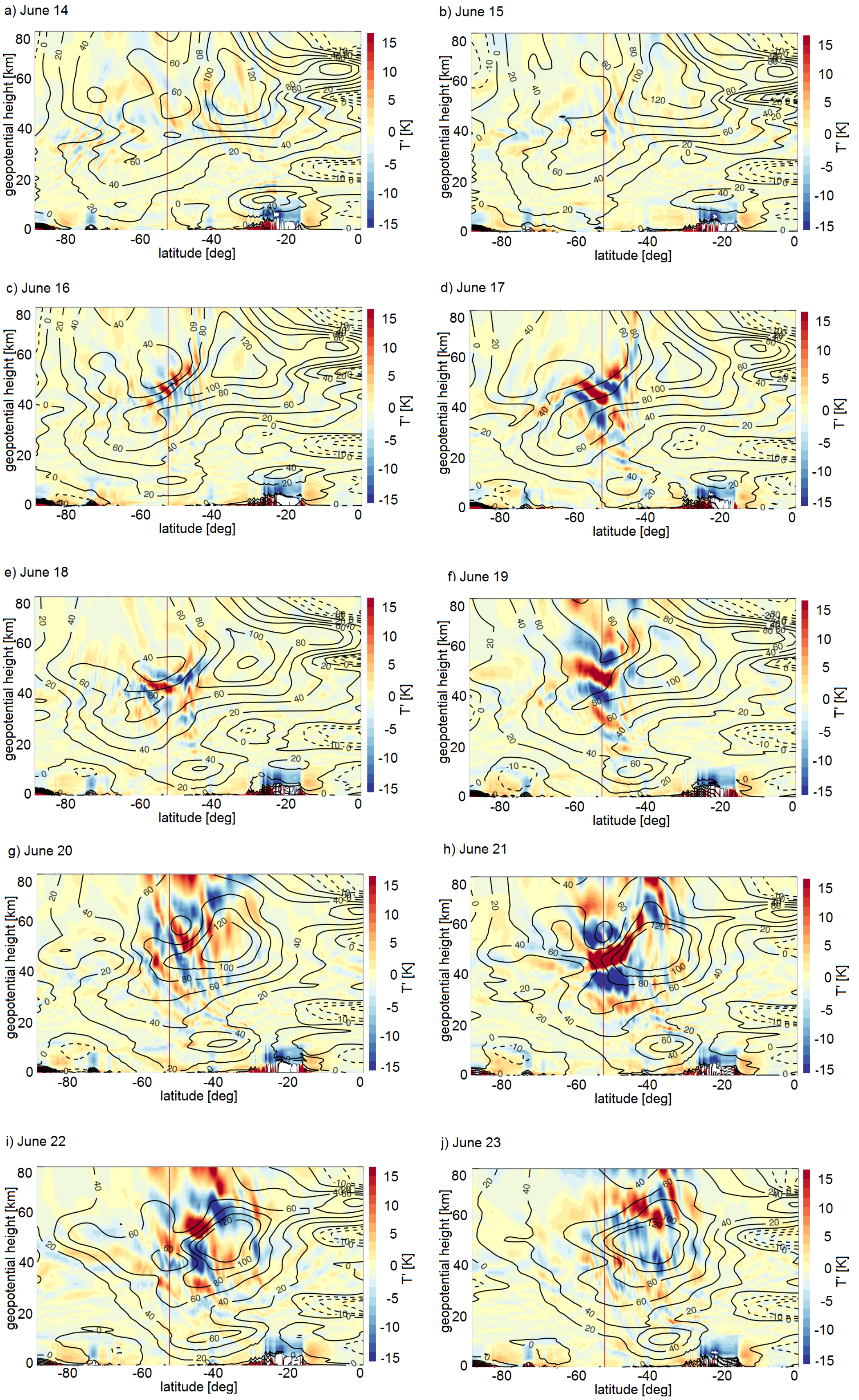

Figure S3. (a-j) Daily IFS latitude-altitude sections at the longitude of Rio Grande (67.75°W) of the zonal wind (contours) and temperature perturbations (color) during 14–23 June 2018, at 0 UT, respectively. The latitude of Rio Grande is indicated by a vertical line.

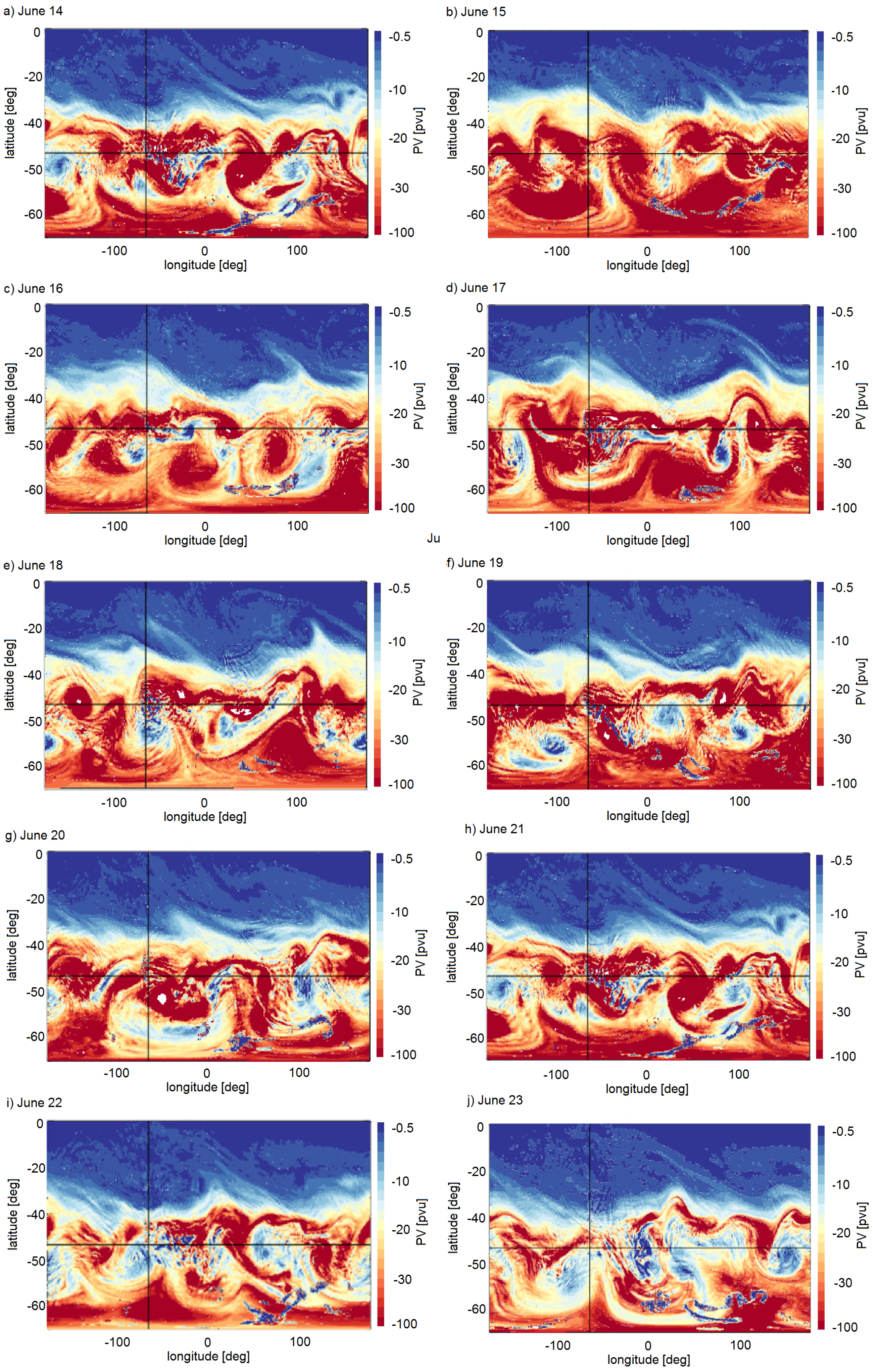

Figure S4. (a-j) Daily IFS potential vorticity maps during 14–23 June 2018, at 0 UT, respectively. The latitude and longitude of Rio Grande is indicated by lines.
